# *Bifidobacterium bifidum* Actively Changes the Gene Expression Profile Induced by *Lactobacillus acidophilus* in Murine Dendritic Cells

**DOI:** 10.1371/journal.pone.0011065

**Published:** 2010-06-10

**Authors:** Gudrun Weiss, Simon Rasmussen, Lisbeth Nielsen Fink, Hanne Jarmer, Birgit Nøhr Nielsen, Hanne Frøkiær

**Affiliations:** 1 Department of Basic Sciences and Environment, Faculty of Life Sciences, University of Copenhagen, Frederiksberg, Denmark; 2 Department of Systems Biology, Center for Biological Sequence Analysis, Technical University of Denmark, Kongens Lyngby, Denmark; Universität Würzburg, Germany

## Abstract

Dendritic cells (DC) play a pivotal regulatory role in activation of both the innate as well as the adaptive immune system by responding to environmental microorganisms. We have previously shown that *Lactobacillus acidophilus* induces a strong production of the pro-inflammatory and Th1 polarizing cytokine IL-12 in DC, whereas bifidobacteria do not induce IL-12 but inhibit the IL-12 production induced by lactobacilli. In the present study, genome-wide microarrays were used to investigate the gene expression pattern of murine DC stimulated with *Lactobacillus acidophilus* NCFM and *Bifidobacterium bifidum* Z9. *L. acidophilus* NCFM strongly induced expression of interferon (IFN)-β, other virus defence genes, and cytokine and chemokine genes related to the innate and the adaptive immune response. By contrast, *B. bifidum* Z9 up-regulated genes encoding cytokines and chemokines related to the innate immune response. Moreover, *B. bifidum* Z9 inhibited the expression of the Th1-promoting genes induced by *L. acidophilus* NCFM and had an additive effect on genes of the innate immune response and Th2 skewing genes. The gene encoding Jun dimerization protein 2 (JDP2), a transcription factor regulating the activation of JNK, was one of the few genes only induced by *B. bifidum* Z9. Neutralization of IFN-β abrogated *L. acidophilus* NCFM-induced expression of Th1-skewing genes, and blocking of the JNK pathway completely inhibited the expression of IFN-β. Our results indicate that *B. bifidum* Z9 actively inhibits the expression of genes related to the adaptive immune system in murine dendritic cells and that JPD2 via blocking of IFN-β plays a central role in this regulatory mechanism.

## Introduction

Dendritic cells (DC) are involved in the initiation of both the innate and adaptive immunity. Pattern recognition receptors expressed on DC and other immune cells contribute to the specific recognition of pathogens as well as harmless microorganisms, e.g. commensals [1], and are thus involved in the subsequent expression of specific genes. Upon encountering of microorganisms, DC orchestrate an innate and, in most cases, a simultaneous adaptive immune response [2], [3].

Probiotic bacteria can be classified in two groups: strong and weak inducers of the pro-inflammatory cytokines IL-12 and tumour necrosis factor (TNF)-α. Co-incubation experiments indicate that there are clear interactions between the signals delivered by bacterial strains. Bifidobacteria, which are weak IL-12 inducers, are able to significantly inhibit the IL-12 production of strong IL-12 inducers like lactobacilli [4], [5]. Interleukin-12-directs a Th1 adaptive immune response, while the induction of a Th2 directed response, although less well-described, is believed to involve DC-derived IL-6 and IL-33 [6], [7]. Almost all lactobacilli and bifidobacteria strains are able to induce significant levels of the anti-inflammatory cytokine IL-10 in DC, however, there are considerable variations in the concentrations measured [5], [8], [9]. The cytokine IL-10 is important for induction of regulatory T cells [10]. Besides the production of cytokines important for the activation of various immune cell subsets, production of chemokines is indispensable for recruitment of specific immune cells. Some chemokines attract in particular T cells, some also monocytes and macrophages, while others specifically recruit cells of the innate immune system, in particular neutrophils [11].

Induction of IL-12 in DC, together with the up-regulation of surface markers such as MHCII, CD86 and CD40, stimulates activation of Th1 cells, but is also important for the production of IFN-γ by NK cells [4]. IL-12 is at least partly induced by interferon (IFN)-β, a key inducer of a strong adaptive immune response against virus infections initiating a signaling cascade that controls the expression of hundreds of interferon-stimulated genes (ISGs) involved in an innate host response against viruses [12]. Several *in vivo* and *in vitro* studies have shown that up-regulation of IFN-β entails a strong Th1 inducing capacity [13]. In DC, IFN-β functions in an autocrine manner through the IFN-α/β receptor (IFNAR) inducing transcription of genes involved in different functions of the cell, such as migration and activation [14]. This includes genes involved in attraction of cells of both the innate and the adaptive immune system (chemokines) and various cytokines, including IL-12 [14]. Whether the transcription of all genes involved in cellular attraction and migration of DC is induced by type I interferons remains to be established.

The precise cellular mechanisms involved in the induction of IFN-β production in DC upon bacterial stimulation are not fully understood. Only recently it has been established that not only vira, but also some bacteria can induce a strong IFN-β production. This includes several Gram-negative bacteria, such as *Salmonella enterica* serovar typhimurium, *Shigella flexneri* and *Escherichia* spp., [15] as well as Gram-positive bacteria, such as *Streptococcus* spp. [16]–[18], and *Listeria monocytogenes* [19], [20]. Upon viral infection, activation of the gene encoding IFN-β critically depends on cooperative interaction of the DNA-binding domains of ATF-2/c-Jun and the immune regulatory factor (IRF)-3 [21]. ATF-2 and c-Jun are members of the large basic-region leucine zipper family of transcription factors, constituting the AP-1 transcription factor complex, which are activated by phosphorylation via mitogen-activated protein kinases (MAPK). Whereas ATF-2 is activated by both the p38 and JNK MAPK, c-Jun is only phosphorylated by JNK [22]. Whether the same mechanism is involved in bacterial induction of IFN-β in DC remains to be described.

The aim of the present work was to elucidate the molecular mechanisms by which *B. bifidum* Z9 is able to inhibit the immune response in murine bone marrow-derived DC initiated by *L. acidophilus* NCFM. Microarrays of DCs stimulated with either *L. acidopilus* NCFM or *B. bifidum* Z9 alone or a mixture of the two bacteria were performed. In DCs incubated with *L. acidophilus* NCFM, genes related to the innate and adaptive immune system triggering a pro-inflammatory and antiviral response were significantly up-regulated. When the two strains were used in combination, *B. bifidum* Z9 exerted both additive and inhibitory effects, as the expression of genes initiating the adaptive immune response induced by *L. acidophilus* NCFM was strongly inhibited, whereas genes of the innate immune response were significantly induced. Our results indicate that the JNK pathway is essential for the expression of IFN-β induced by a bacterial stimulus. In this context, the gene encoding Jun dimerization protein 2 (JDP2) was only up-regulated in cells stimulated with *B. bifidum* Z9 and is therefore a candidate gene for the inhibition of IFN-β transcription in *L. acidophilus* NCFM stimulated DC.

## Materials and Methods

### Bacterial Strains, Growth Conditions and Preparation of UV-killed Bacteria

The strains *Lactobacillus acidophilus* NCFM (Danisco, Copenhagen, Denmark) and *Bifidobacterium bifidum* Z9 (Copenhagen University, Faculty of Life Sciences, Denmark) were grown anaerobically overnight at 37°C in de Man Rogosa Sharp (MRS) broth (Merck, Darmstadt, Germany) and sub-cultured twice. Cells were harvested by centrifugation at 2,000×g for 15 min, washed twice in phosphate-buffered saline (PBS, Cambrex Bio Whittaker, East Rutherford, NJ, USA) and re-suspended in 1/10 the growth volume of PBS. As UV-killed bacteria display the same stimulatory pattern as live bacteria, UV-killed bacteria were used to avoid the risk of growth during stimulation of DC and to secure high repeatability by the use of the same batch of bacteria. The bacteria were killed by 20 min exposure to UV light and stored at −80°C. Killing was verified by plating the UV-exposed bacteria on MRS plates. To determine the bacterial concentration, 3 ml of the preparations were lyophilised as triplicates and the dry weight was corrected for buffer salt content.

### Generation of Murine Dendritic Cells

Bone marrow-derived DCs were prepared as described previously [23]. Briefly, bone marrow from three male C57BL/6 mice (Tactonic, Lille Skensved, Denmark) was flushed out from the femur and tibia and washed. 3×10^5^/ml bone marrow cells were seeded into 10 cm Petri dishes in 10 ml RPMI 1640 (Sigma-Aldrich, St. Louis, MO, USA) containing 10% (v/v) heat-inactivated fetal calf serum supplemented with penicillin (100 U/ml), streptomycin (100 µg/ml), glutamine (4 mM), 50 µm 2-mercaptoethanol (all purchased from Cambrex Bio Whittaker) and 15 ng/ml murine GM-CSF (harvested from a GM-CSF transfected Ag8.653 myeloma cell line). The cells were incubated for 8 days at 37°C in 5% CO_2_ humidified atmosphere. On day 3, 10 ml of complete medium containing 15 ng/ml GM-CSF was added. On day 6, 10 ml were removed and replaced by fresh medium. Non-adherent, immature DC were harvested on day 8.

### Stimulation of Murine Dendritic Cells with Bacteria

Immature DC (2×10^6^ cells/ml) were resuspended in fresh medium supplemented with 10 ng/ml GM-CSF, and 500 µl/well were seeded in 48-well tissue culture plates (Nunc, Roskilde, Denmark). *Lactobacillus acidophilus* NCFM was added in a final concentration of 10 µg/ml and *Bifidobacterium bifidum* Z9 in a final concentration of 40 µg/ml (both individually and in combination; total volume 100 µl/well). Optimal bacterial concentrations were determined in a previous study [24]. The cell cultures were incubated at 37°C in 5% CO_2_. In the MAPK inhibitor experiments, DC (2×10^6^ cells/ml) were pre-incubated for 1 h with SP600125, a specific inhibitor of JNK1/2 (Invivogen, San Diego, CA, USA), SB203580, a specific inhibitor of p39 MAPK (Invivogen), and the MEK1/2 inhibitor U0126 which blocks MEK1/2 and thereby phosphorylation of the target ERK1/2 (Cell Signaling, MA, USA). For the IFN-β inhibition assay, mouse IFN-β polyclonal antibody (R&D Systems, Minneapolis, USA) was added to the DC (10 µg/ml and 50 µg/ml) immediately after addition of *L. acidophilus* NCFM (10 µg/ml). Unless indicated otherwise, DC were harvested for RNA extraction after 10 h and the supernatant for ELISA analysis after 20 h.

### RNA Extraction

Murine DC were harvested after 4 h and 10 h of stimulation, homogenised by QIAshredder (Qiagen, Ballerup, Denmark), and RNA was extracted using the RNeasy Plus Mini Kit (Qiagen). RNA quality was verified by Bioanalyzer (Agilent, Santa Clara, USA), and the concentration was determined by Nanodrop (Thermo, Wilmington, USA).

### Microarray Analysis

RNA was extracted per stimulation representing DC from three C57BL/6 mice incubated for 10 h with either *Lactobacillus acidophilus* NCFM or *Bifidobacterium bifidum* Z9 or with both bacteria in combination. RNA from unstimulated DC was included as a negative control. 1 µg RNA was converted into cDNA, and biotin-labeled aRNA was synthesized using the MessageAmp™ II-Biotin Enhanced Kit (Ambion, Austin, TX, USA) according to the manufacturer's instructions. The Gene Chip Mouse genome 430 2.0 Array (Affymetrix, Santa Clara, CA, USA) comprising 45,000 probe sets representing over 34,000 mouse genes was applied. The arrays were stained, washed and scanned according to the manufacturer's instructions. The microarray data was analyzed using R and Bioconductor [25]. Raw probe intensities were normalized using *qspline* and expression index calculations were performed using *rma* [26], [27]. For statistical testing, a two-way ANOVA was applied and the false discovery rate (FDR) was estimated using a Monte Carlo approach. The statistical significance was set at an FDR of 0 yielding 1924, 1264 and 490 significantly up-regulated genes (Ensembl 56) upon stimulation with *L. acidophilus NCFM* and *B. bifidum Z9* and both strains combined, respectively. Gene Set Enrichment (GSEA) was performed applying the method described by Kim and Volsky [28] in R using the *PGSEA* package (Kyle Furge and Karl Dykema (2006). PGSEA: Parametric Gene Set Enrichment Analysis, R package version 1.10.0). For this analysis only genes with an absolute fold change greater than 2 were included.

### Quantitative Real Time PCR Analysis

1 µg of total RNA was reverse transcribed by the TaqMan Reverse Transcription Reagent kit (Applied Biosystems, Foster City, USA) using random hexamer primers according to the manufacturer's instructions. The obtained cDNA was stored in aliquots at −80°C. The following TaqMan Gene Expression Assays were purchased (Applied Biosystems): Cxcl1 (Assay ID Mm00433859_m1), Cxcl2 (Assay ID Mm00436450_m1), Cxcl10 (Assay ID Mm00445235_m1) and Ccl12 (Assay ID Mm01617100_m1), IL1b (Assay ID Mm00434228_m1). For the selection of other primers and probes, the regions coding for the genes investigated were retrieved from the GenBank and EMBL databases. The following gene sequences were applied: IFN-β (NM_010510), IL-12 p40 (NM_008352), IL-10 (NM_010548) and beta actin (NM_007393). Primers and probes were designed using the software Primer Express 3.0 (Applied Biosystems) and tested for specificity by the basic alignment search tool BLAST. HPLC purified forward and reverse primers were manufactured by DNA Technology (Aarhus, Denmark). The probes were labelled with the 5′ reporter dye 6-carboxy-fluorescein (FAM) and the 3′ quencher dye NFQ-MGB (Applied Biosystems). Sequences of primers and probes are listed in Table 1. Primer and probe concentrations were optimized and to determine the efficiency of the amplifications, dilution standard curves were made for each set of primers and probe (data not shown). The amplifications were carried out in a total volume of 20 µl containing 1×TaqMan Universal PCR Master Mix (Applied Biosystems), forward and reverse primer (concentration 900 nM each), 200 nM TaqMan MGB probe, and purified target cDNA. The cycling parameters were initiated by 20 sec at 95°C, followed by 40 cycles of 3 sec at 95°C and 30 sec at 60°C using the ABI Prism 7500 (Applied Biosystems). Amplification reactions were performed in triplicate, and DNA contamination controls were included. The amplifications were normalised to the expression of the beta actin encoding gene. Relative transcript levels were calculated applying the equation described by Pfaffl *et al.* [29].

**Table 1:** Primers and Probes used for Real-Time PCR Analysis.

Target	Primers and Probes	Sequence (5′-3′)
IFN-β (NM_010510)	Forward	CGGACTTCAAGATCCCTATGGA
	Reverse	TGGCAAAGGCAGTGTAACTCTTC
	Probe	ATGACGGAGAAGATGC
IL-12 p40 (NM_008352)	Forward	TGGAGCACTCCCCATTCCT
	Reverse	TGCGCTGGATTCGAACAA
	Probe	CTTCTCCCTCAAGTTC
IL-10 (NM_010548)	Forward	GATGCCCCAGGCAGAGAA
	Reverse	CACCCAGGGAATTCAAATGC
	Probe	CATGGCCCAGAAAT
Beta actin (NM_007393)	Forward	CGATGCCCTGAGGCTCTTT
	Reverse	TGGATGCCACAGGATTCCA
	Probe	CCAGCCTTCCTTCTT

### Cytokine Quantification by ELISA

The production of murine IFN-β, IL-12(p70), IL-10, IL-6, IL-1β and TNF-α was analysed using commercially available enzyme-linked immunosorbent assay kits (R&D Systems, Minneapolis, USA).

### Statistical Analysis

One-way ANOVA and Bonferroni tests were applied to analyse the real-time PCR gene expression data. Statistical calculations were performed using the software program GraphPad Prism 5 (La Jolla, CA, USA).

## Results

### Inhibitory and Additive Effects of *B. bifidum* Z9 on the Gene Expression in Murine Dendritic Cells are Time dependent

**Figure 1:**
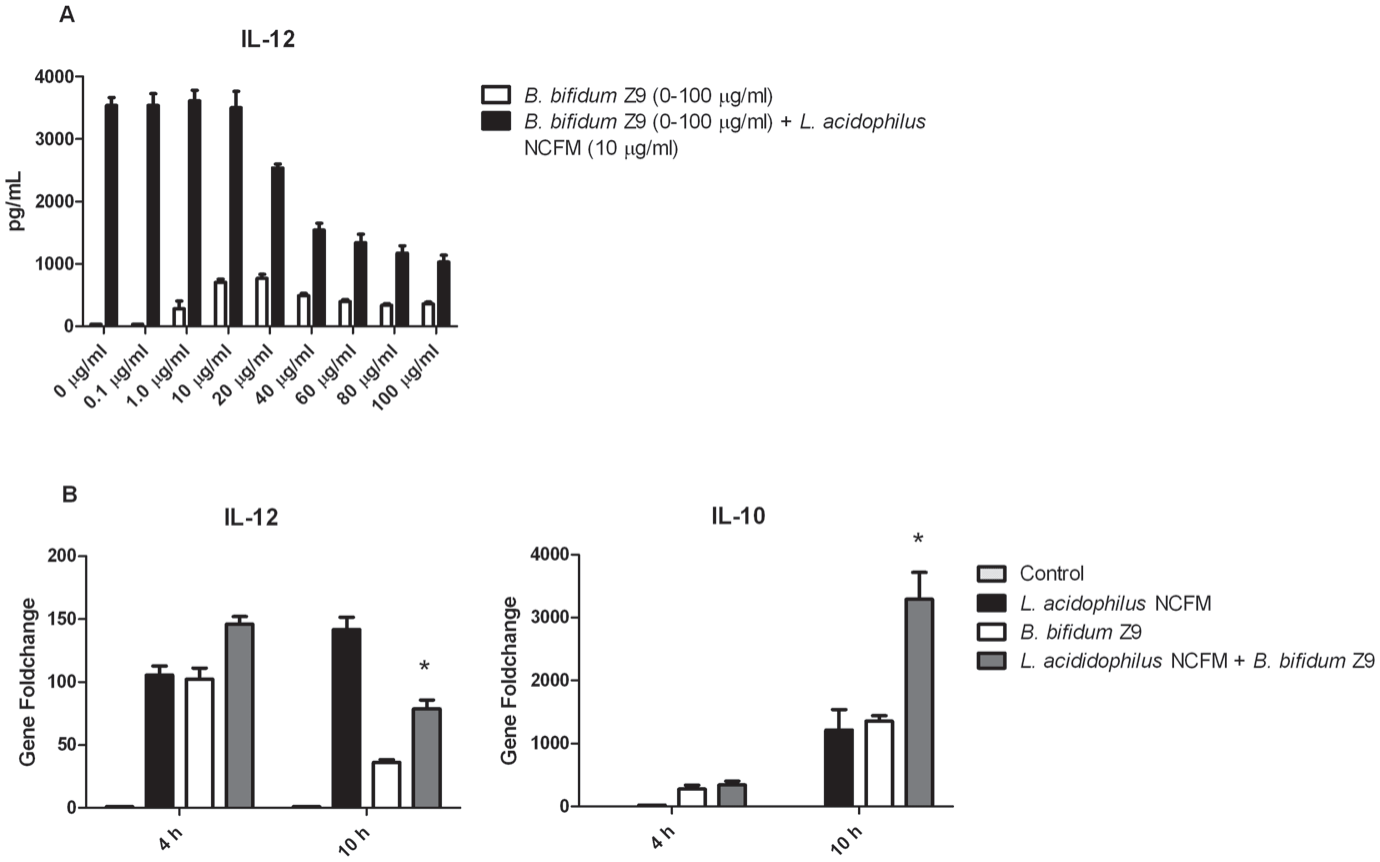
Inhibitory and additive effects of *Bifidobacterium bifidum* Z9. **A.** The inhibitory effect of *B. bifidum* Z9 on the IL-12 production of *L. acidophilus* NCFM stimulated dendritic cells was determined by ELISA testing various concentrations of *B. bifidum* Z9 (0–100 µg/ml) against a constant concentration of *L. acidophilus NCFM* (10 µg/ml). **B.** *B. bifidum* Z9 exerts inhibitory effects on the expression of *Il12* and additive effects on the expression of *Il10* in *L. acidophilus* NCFM stimulated dendritic cells in a time dependent manner. Gene expression was measured by RT-PCR 4 h and 10 h after stimulation with the indicated bacteria (*L. acidophilus* NCFM versus *L. acidophilus* NCFM+*B. bifidum* Z9 *P*<0.01). Data show results from one out of five independent experiments.

Our previous finding that some probiotic bacteria strains can inhibit the stimulatory effects of others led us to compare the gene expression of DC stimulated with both *L. acidophilus* NCFM and *B. bifidum* Z9 simultaneously. To determine the optimal inhibitory effect on the IL-12 production, *B. bifidum* Z9 was added in increasing concentrations from 0–100 µg/ml to DC stimulated with 10 µg/ml *L. acidophilus* NCFM (Figure 1A). The combination of 40 µg/ml *B. bifidum* Z9 and 10 µg/ml *L. acidophilus* NCFM inhibited the IL-12 production by 69% and was used in all further experiments. Figure 1B demonstrates that *B. bifidum* Z9 exerts inhibitory and additive effects on the gene expression in murine DC stimulated with *L. acidophilus* NCFM in a time dependent manner. A distinct up-regulation of the pro-inflammatory cytokine *Il12* was detected in murine DC after 4 h of incubation with *L. acidophilus* NCFM and *B. bifidum* Z9, as well as with both strains in combination. However, whereas the expression of *Il12* decreased in *B. bifidum* Z9 stimulated DC at 10 h, it increased in *L. acidophilus* NCFM stimulated cells. In DC stimulated with both strains, *Il-12* was down-regulated by 45% at 10 h compared to cells stimulated with *L. acidophilus* NCFM alone. In the case of the anti-inflammatory cytokine *Il10*, a distinct up-regulation in DC upon stimulation with *L. acidophilus* NCFM and *B. bifidum* Z9 was first detected at 10 h. With both strains in combination, a significant additive effect on *Il10* (171%) compared to DC stimulated with *L acidophilus* NCFM alone was detected at 10 h.

### Microarray Analysis reveals Induction of Differential Groups of Genes by *L. acidophilus* NCFM and *B. bifidum* Z9

**Figure 2:**
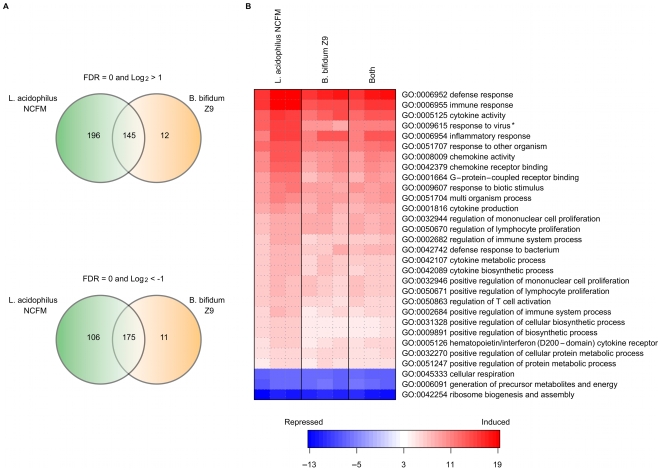
Microarray analysis of dendritic cells stimulated with *Lactobacillus acidophilus* NCFM and *Bifidobacterium bifidum* Z9. Up- and downregulation of genes upon incubation of dendritic cells with *L. acidophilus* NCFM and *B. bifidum* Z9, respectively, and both strains in combination. A. Venn diagram depicting the distinct up- and down regulation and overlap of genes. Only genes with an absolute fold change greater than 2 were included. B. Parametric Gene Set Enrichment of Gene Ontology (GO) Biological Process terms of the top 30 most significant GO terms comparing *L. acidophilus* NCFM vs. control (no stimulation). Blue indicates repression of the genes, white indicates no change and red indicates induction of the genes in the GO term. Only the GO term “response to virus” was significantly different (*) when comparing *L. acidophilus NCFM* and *B. bifidum Z9* responses.

To further analyze the differences in gene expression in DC upon stimulation with either of the two bacteria or both strains in combination, we performed genome wide microarray analysis of DC harvested 10 h after stimulation. 341 genes were significantly up-regulated by more than two-fold upon *L. acidophilus* NCFM stimulation, 157 upon *B. bifidum* Z9 stimulation, and of these, 145 genes were common for both strains (Figure 2). Likewise, 281 genes were significantly down-regulated by more than two fold upon stimulation with *L. acidophilus* NCFM, 186 genes upon stimulation with *B. bifidum* Z9 and of these, 175 genes were induced in DC stimulated with both strains simultaneously. The data generated are deposited in NCBI's Gene Expression Omnibus (PMID: 11752295) and are accessible through GEO Series accession number GSE20302. A Parametric Gene Set Enrichment (PGSEA) of GO terms revealed that the responses of the DCs upon stimulation with *L. acidophilus* NCFM and *B. bifidum* Z9 were similar (Fig. 2B). “Response to virus” was the only GO term enrichment significantly higher up-regulated (P-value = 0.003) in DC stimulated with *L. acidophilus* NCFM compared to DC stimulated with *B. bifidum* Z9. Detailed study of the expression of individual genes revealed that *L. acidophilus* NCFM induced a pro-inflammatory response with a Th1 skewing profile. By contrast, *B. bifidum* Z9 induced a more Th2 skewed response and mainly up-regulated genes related to the innate immune response (Table 2, 3, 4 and 5).

**Table 2:** Inhibitory Effects of *Bifidobacterium bifidum* Z9 on the Chemokine Expression in *Lactobacillus acidophilus* NCFM stimulated Dendritic Cells.

Ensembl Gene Nr.	Annotation	*L. acidophilus*	*B. bifidum*	*L. acidophilus *+ *B. bifidum*	Name
00000029417	*Cxcl9*	4.0	1.8	2.2	CXC ligand 9
00000034855	*Cxcl10*	5.2	2.9	4.3	CXC ligand 10
00000060183	*Cxcl11*	3.6	0.96	2.2	CXC ligand 11
00000000982	*Ccl3*	1.0	0.61	0.75	CC ligand 3
00000018930	*Ccl4*	1.1	0.075	0.63	CC ligand 4
00000035373	*Ccl7*	2.8	1.5	2.3	CC ligand 7
00000035352	*Ccl12*	2.8	0.86	1.0	CC ligand 12

Data indicates gene expression fold changes of dendritic cells stimulated with *L. acidophilus* NCFM, *B. bifidum* Z9 and both strains in combination in comparison with un-stimulated dendritic cells measured on Affymetrix microarrays. Foldchanges are log_2_ relative to control.

**Table 3:** Additive Effects of *Bifidobacterium bifidum* Z9 on the Chemokine Expression in *Lactobacillus acidophilus* NCFM stimulated Dendritic Cells.

Ensembl Gene Nr.	Annotation	*L. acidophilus*	*B. bifidum*	*L. acidophilus *+ *B. bifidum*	Name
00000029380	*Cxcl1*	3.2	4.5	4.7	CXC ligand 1
00000058427	*Cxcl2*	1.7	2.3	2.2	CXC ligand 2
00000029371	*Cxcl5*	3.5	4.2	4.4	CXC ligand 5

Data indicates gene expression fold changes of dendritic cells stimulated with *L. acidophilus* NCFM, *B. bifidum* Z9 and both strains in combination in comparison with un-stimulated dendritic cells measured on Affymetrix microarrays. Foldchanges are log_2_ relative to control.

**Table 4:** Inhibitory Effects of *Bifidobacterium bifidum* Z9 on the Cytokine Expression in *Lactobacillus acidophilus* NCFM stimulated Dendritic Cells.

Ensembl Gene Nr	Annotation	*L. acidophilus*	*B. bifidum*	*L. acidophilus *+ *B. bifidum*	Name
00000048806	*Ifnb1*	2.5	0.9	0.48	Interferon beta 1
00000004296	*Il12b*	5.5	4.3	5	Interleukin 12b
00000031712	*Il15*	2.7	1.8	2.4	Interleukin 15
00000024401	*Tnf*	2.1	1.3	1.7	Tumor necrosis factor alpha
00000039217	*Il18*	2.0	1.0	1.4	Interleukin 18
00000025383	*Il23a*	1.0	0.37	0.68	Interleukin 23a
00000027776	*Il12a*	0.77	0.6	0.59	Interleukin 12a

Data indicates gene expression fold changes of dendritic cells stimulated with *L. acidophilus* NCFM, *B. bifidum* Z9 and both strains in combination in comparison with un-stimulated dendritic cells measured on Affymetrix microarrays. Foldchanges are log_2_ relative to control.

**Table 5:** Additive Effects of *Bifidobacterium bifidum* Z9 on the Cytokine Expression in *Lactobacillus acidophilus* NCFM stimulated Dendritic Cells.

Ensembl Gene Nr	Annotation	*L. acidophilus*	*B. bifidum*	*L. acidophilus *+ *B. bifidum*	Name
00000016529	*Il10*	2.5	3.0	3.6	Interleukin 10
00000025746	*Il6*	4.0	5.0	5.1	Interleukin 6
00000027399	*Il1a*	2.2	2.3	2.4	Interleukin 1 alpha
00000027398	*Il1b*	2.1	2.3	2.3	Interleukin 1 beta
00000024810	*Il33*	0.76	1.8	2.3	Interleukin 33

Data indicates gene expression fold changes of dendritic cells stimulated with *L. acidophilus* NCFM, *B. bifidum* Z9 and both strains in combination in comparison with un-stimulated dendritic cells measured on Affymetrix microarrays. Foldchanges are log_2_ relative to control.

One of the main differences observed comparing the stimulatory patterns of *L. acidophilus* NCFM and *B. bifidum* Z9 was the induction of chemokine encoding genes (Table 2 and 3). A clear trend towards an up-regulation of chemokines involved in attraction of Th1 cells (*Cxcl9*, *Cxcl10* and *Cxcl11*) and/or monocytes/macrophages (*Ccl3*, *Ccl4*, *Ccl7* and *Ccl12*) upon stimulation with *L. acidophilus* NCFM emerged, whereas *B. bifidum* Z9 gave rise to a much lower gene induction of Th1 recruiting chemokines (55% reduction of *Cxcl9*, 44% reduction of *Cxcl10* and 73% reduction of *Cxcl11* compared to DC stimulated with *L. acidophilus NCFM*). The gene expression of the monocytes/macrophages attracting chemokines was likewise much lower, from no up-regulation (*Ccl4*) to approximately 50% of the up-regulation (*Ccl3*, *Ccl4* and *Ccl7*) detected after stimulation with *L. acidophilus*. In contrast, stimulation of DC with *B. bifidum* Z9 resulted in the highest up-regulation of chemokines involved in the attraction of neutrophils (*Cxcl1*, *Cxcl2*, and *Cxcl5*), although these genes were also up-regulated, yet to a lower extent, in *L. acidophilus* NCFM stimulated cells (70–80% up-regulation compared to *B. bifidum* Z9 stimulated cells).

**Figure 3:**
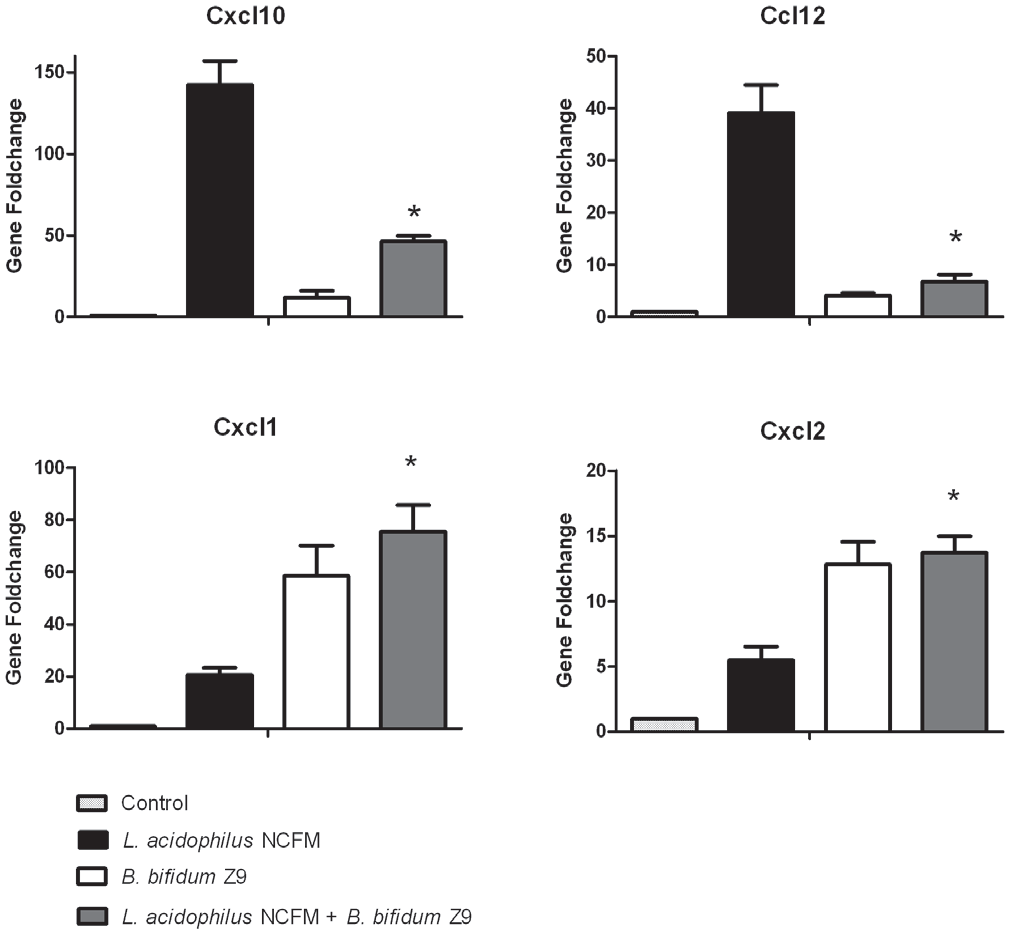
Induction of chemokines in *Lactobacillus acidophilus* NCFM and *Bifidobacterium bifidum* Z9 stimulated dendritic cells. Gene expression analysis of the chemokines *Cxcl10*, *Ccl12*, *Cxcl1* and *Cxcl2* in dendritic cells upon stimulation with *L. acidophilus* NCFM, *B. bifidum* Z9 or the two bacteria in combination and unstimulated dendritic cells (control) was performed by RT-PCR after 10 h of stimulation (*L. acidophilus* NCFM+*B. bifidum* Z9 versus *L. acidophilus* NCFM, *P*<0.01).

When both strains were added in combination, *B. bifidum* Z9 exerted inhibitory effects on the expression of the Th1 attracting chemokines induced by *L. acidophilus* NCFM (*Cxcl9*, *Cxcl10* and *Cxcl11*) and additive effects on the neutrophil recruiting chemokines mostly induced by *B. bifidum* Z9 (*Cxcl1*, *Cxcl2*, and *Cxcl5*). It can be concluded that co-stimulation of DC with *L. acidophilus* NCFM and *B. bifidum* Z9 has a significant inhibitory effect on the gene expression of chemokines leading to an abrogation of the pro-inflammatory and Th1 polarizing response induced by *L. acidophilus* NCFM. In analogy with the neutrophil attracting chemokine profiles, *B. bifidum* Z9 strongly induced, and furthermore had an additive effect on the *L. acidophilus* NCFM-induced expression of the gene encoding granulocyte colony-stimulating factor (G-CSF), which is important for the proliferation and differentiation of neutrophil precursors and mature neutrophils [30] (data not shown). Gene expression results obtained by microarray analysis were confirmed for *Cxcl10*, *Ccl12*, *Cxcl1* and *Cxcl2* by Real-Time PCR (Figure 3). With both strains in combination, *B. bifidum* Z9 significantly down-regulated the *L. acidophilus* NCFM induced expression of the Th1 attracting chemokine *Cxcl10* by 90% and the expression of the monocyte and macrophage recruiting chemokine *Ccl12* by 85%, whereas the effect on the *Cxcl1* chemokine was additive (30% induction in comparison to *B. bifidum* Z9 alone). With both strains in combination, the chemokine *Cxcl2* was expressed at a lower level which is comparable to the level of *Cxcl2* expressed by DC stimulated with *B. bifidum* Z9 alone.

Also regarding the expression of cytokine genes, a clear distinction between the two stimulation regimes was observed (Table 4 and 5). The pro-inflammatory and/or Th1 inducing cytokine genes *Ifnb1*, *Il12*, *Il18*, and *Tnf* were strongly up-regulated only in *L. acidophilus* NCFM stimulated DC, whereas *B. bifidum* Z9 strongly up-regulated the cytokines *Il10*, *Il15*, *Il1b* and *Il33*. Thus, when DC were stimulated with both strains simultaneously, the inhibitory pattern was similar to the one observed for chemokines, as the pro-inflammatory cytokine genes induced by *L. acidophilus* NCFM were significantly down-regulated. The most definite inhibitory effect of *B. bifidum* Z9 was detected for the gene encoding IFN-β, as it was down-regulated by 81%. In contrast, simultaneous stimulation with the two bacteria led to a higher expression of *Il10*, *Il15*, *Il1b* and *Il33* compared to DC stimulated with *B. bifidum* Z9 alone. The strongest additive effect upon co-stimulation with the two bacteria was detected for *Il33*, a cytokine important for the activation of NFkB, MAPK kinases, and the production of Th2-associated cytokines [7].

**Figure 4:**
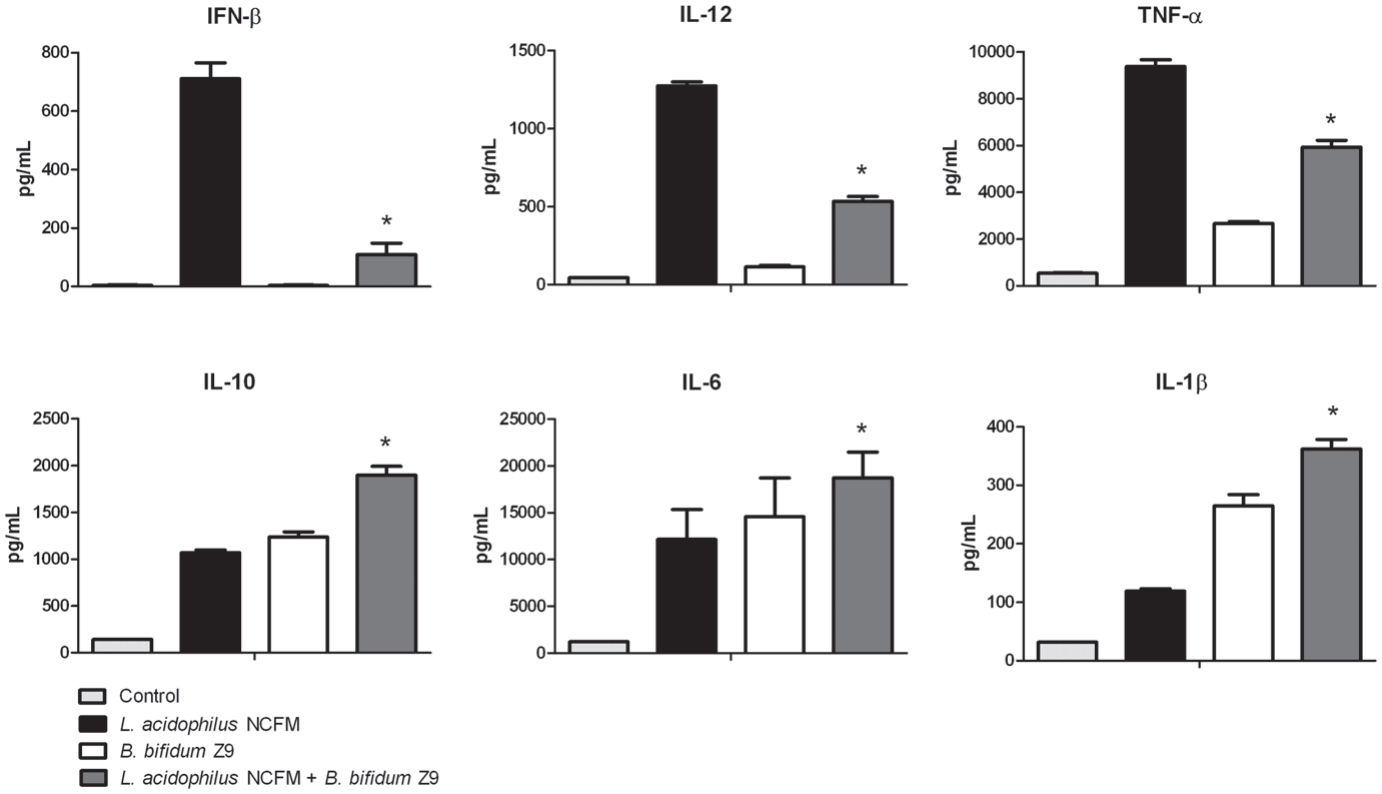
Induction of cytokines in *Lactobacillus acidophilus* NCFM and *Bifidobacterium bifidum* Z9 stimulated dendritic cells. Protein expression of cytokines was measured by ELISA in supernatants from dendritic cells after 20 h of stimulation with *L. acidophilus* NCFM, *B. bifidum* Z9, or with both strains in combination. Supernatant from unstimulated DC were used as controls (*L. acidophilus* NCFM+*B. bifidum* Z9 versus *L. acidophilus* NCFM *P*<0.01). Results are representative of 2 experiments.

Interestingly, microarray analysis revealed that only *B. bifidum* Z9 was able to down-regulate the expression of the genes induced by *L. acidophilus* NCFM in murine DC. This effect was not observed vice versa. The induction of the cytokines *Ifnb1*, *Il12*, *Il10* and *Ilβ1* was confirmed both on gene level by RT-PCR (data not shown) and protein level by ELISA (Figure 4).

### IFN-β is a Key Inducer of the Pro-inflammatory and Th1 Polarizing Response

**Figure 5:**
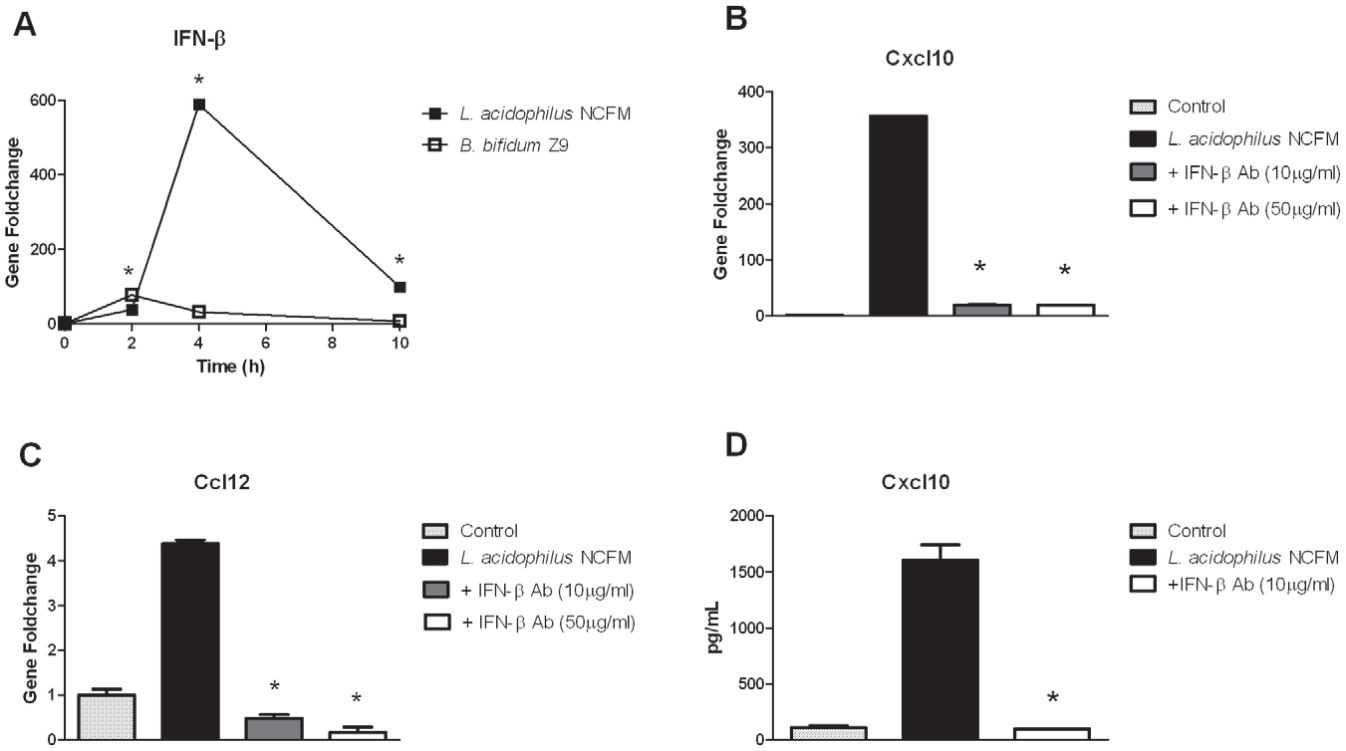
*Lactobacillus acidophilus* NCFM induces IFN-β in dendritic cells. The IFN-β encoding gene is strongly up-regulated in *L .acidophilus NCFM* stimulated dendritic cells and responsible for the expression of the chemokines Cxcl10 and Ccl12. **A**. Expression of the IFN-β encoding gene over time in dendritic cells upon stimulation with *L. acidophilus* NCFM and *B. bifidum* Z9. **B**. Inhibition of IFN-β activity induced by *L. acidophilus* NCFM by addition of polyclonal IFN-β antibodies measured via the gene expression of the chemokine *Cxcl10* after 10 h and **C**. via the gene expression of the chemokine *Ccl12* after 10 h and **D**. via protein production of Cxcl10 in the supernatant after 24 h of stimulation by ELISA (*P*<0.01).

Upon stimulation of DC, *L. acidophilus* NCFM induced a distinct up-regulation of IFN-β, the key inducer of anti-viral responses, leading to the activation of multiple interferon-stimulated genes (ISGs) involved in the innate host response against viruses. We therefore hypothesized that the onset of the pro-inflammatory response initiated by *L. acidophilus* NCFM is dependent on IFN-β. We determined the expression of the gene encoding IFN-β over time (2 h, 4 h and 10 h) in DC stimulated with *L. acidophilus* NCFM and *B. bifidum* Z9, respectively (Figure 5A). When DC were incubated with *L. acidophilus* NCFM, the gene encoding IFN-β was only slightly up-regulated after 2 h (38-fold), but reached a significant maximum after 4 h (589-fold) that declined to 100-fold after 10 h. By contrast, stimulation of DC with *B. bifidum Z9* only gave rise to a slight up-regulation of the gene encoding IFN-β at 2 h, which was down-regulated again after 4 h. To investigate whether the induction of the chemokines *Cxcl10* and *Ccl12* is dependent on IFN-β, we added polyclonal anti-IFN-β antibodies to the DC immediately after addition of *L. acidophilus* NCFM and measured the gene expression profiles of *Cxcl10* and *Ccl12* at 10 h. In the case of the chemokine *Cxcl10*, the very strong expression of the gene was completely abrogated when polyclonal anti-IFN-β antibodies were added (Figure 5B). The same pattern was observed for the gene encoding the chemokine Ccl12 (Figure 5C). The inhibition of the expression of *Cxcl10* was confirmed on the protein level by ELISA, as the addition of 10 µg/ml polyclonal IFN-β antibodies completely inhibited the protein production of Cxcl10 (Figure 5D).

### *B. bifidum* Z9 induces the Expression of Jun dimerization protein 2 (*Jdp2*)

**Figure 6:**
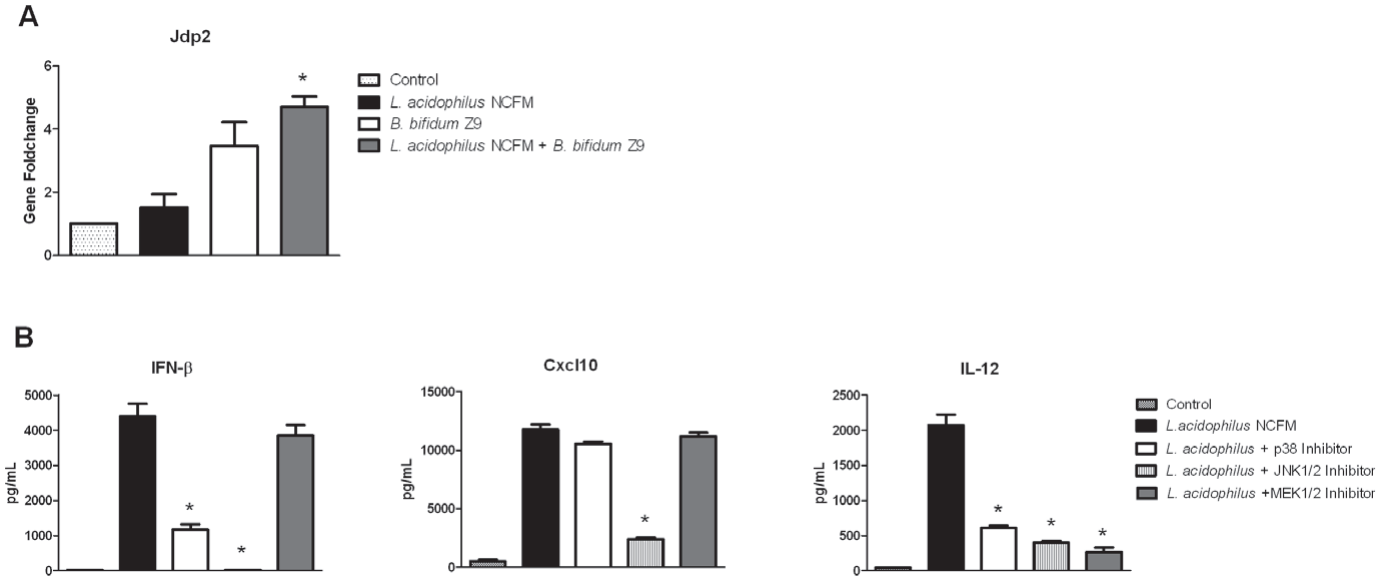
*Jdp2* and MAPK kinases control immune responses in dendritic cells. Expression of *Jdp2* and inhibition of MAPK kinases in dendritic cells upon stimulation of *L. acidophilus* NCFM. **A**. *B. bifidum* Z9 exerts an additive effect on the expression of *Jdp2* in DC stimulated with *L. acidophilus* NCFM at timepoint 10 h measured by RT-PCR (*L. acidophilus* NCFM+*B. bifidum* Z9 versus *L. acidophilus* NCFM, *P*<0.01). **B**: Protein expression of IFN-β, Cxcl10 and IL-12 in murine *L. acidophilus* NCFM stimulated dendritic cells measured by ELISA in the presence of the MAPK inhibitors p38, JNK1/2 and MEK1/2 (*L. acidophilus* NCFM versus inhibitors, *P*<0.01).

Our results suggest that *B. bifidum* Z9 inhibits the up-regulation of genes induced by *L. acidophilus* NCFM by actively leading to an expression of genes involved in the repression of IFN-β. Microarray analysis revealed that only few genes were significantly induced by *B. bifidum* Z9. One of these genes, which encodes Jun dimerization protein 2 (JDP2), is of special interest. JDP2 is a basic leucine zipper transcription factor family member that interacts with the transcription factors c-Jun and ATF2, leading to inhibition of their transcription-promoting activities, and thus plays a major role in the regulation of cell signaling. In contrast to homodimers of c-Jun and heterodimers of c-Jun and ATF2, JDP2 forms heterodimers with c-Jun and ATF2 and acts as a repressor at the AP-1 site [31]. Thus, JDP2 blocks the transcription of genes otherwise expressed upon MAPK phosphorylaton of c-Jun and other AP-1 subunits. Our microarray analysis revealed that *Jpd2* was induced in DC upon stimulation with *B. bifidum* Z9 (1.6-fold) and with both strains in combination (1.8-fold), but to a much lower extent upon stimulation with *L. acidophilus* NCFM alone (0.49-fold). This induction of *Jpd2* was verified by RT-PCR (Fig 6A).

### MAPK JNK Kinase directs the Activation of the Immune System in *L. acidophilus* NCFM stimulated Dendritic Cells

As the induction of *Jdp2* may be implicated in the suppression of IFN-β and other cytokines up-regulated by *L. acidophilus* NCFM, but down-regulated by *B. bifidum* Z9, we hypothesized that the MAP kinases p38 and JNK, but not ERK, are involved in the expression of these cytokines. Our next approach was therefore to use MAPK kinase inhibitors (blocking MAPK p38, JNK1/2, and MEK1/2) to test this hypothesis. As demonstrated in figure 6B, blocking of the JNK1/2 pathway completely inhibited the protein expression of IFN-β in DC stimulated with *L. acidophilus* NCFM, whereas blocking of p38 only resulted in a partial inhibition (73%). Blocking of ERK phosphorylation by the MEK1/2 inhibitor did not affect the IFN-β expression induced by *L. acidophilus* NCFM. The same pattern emerged for Cxcl10 (Fig. 6B) as well as for TNF-α (data not shown). The production of IL-12 was significantly inhibited by all 3 MAPK kinase inhibitors (Fig. 6B). Hence, as JNK activates the transcription factors c-Jun and ATF-2 and is a prerequisite for the production of IFN-β, the up-regulation of *Jdp2* is most likely to affect the activity of c-Jun and ATF-2. This, in turn, has an impact on the expression of IFN-β through its binding to their common response element.

We therefore suggest that *B. bifidum* Z9 actively inhibits the induction of genes in DC triggering the adaptive immune system and that JDP2 plays a major role in the regulatory mechanism.

## Discussion

It has previously been shown by us and other groups that different bacteria considered as beneficial for our health have highly varying effects on the cells of the immune system, including DC, and that a combination of bacteria exerts inhibitory or additive effects [4], [5], [9], [23]. In the present study we used genome wide microarray analysis to further explore this phenomenon and to elucidate the underlying cellular mechanisms.

First of all, our results indicate that the gene expression after stimulation is time dependent.

At 4 h, the expression of the cytokine *Il12* was similar for the two bacteria, whereas the expression increased strongly in *L. acidophilus* NCFM stimulated DC but decreased in *B. bifidum* Z9 stimulated DC. This indicates that activation of kinases may give rise to initial production of IFN-β and IL-12 in the *B. bifidum* Z9 stimulated cells, which is subsequently inhibited by newly expressed genes involved in a down-regulating mechanism. The microarray analysis revealed that far more genes were up-regulated in *L. acidophilus* NCFM stimulated DC than in *B. bifidum* Z9 stimulated DC. Most of these genes induced by *L. acidophilus* NCFM were down-regulated when both bacteria were added in combination. Thus, our earlier findings regarding the differences in production of the pro-inflammatory cytokines IL-12 and TNF-α in DCs stimulated with either of the two bacterial genera [5] can be extended to account for a high number of genes. A gene set enrichment analysis of GO terms, however, only revealed that genes related to virus defence were significantly higher up-regulated after stimulation of DC with *L. acidophilus* NCFM compared to stimulation with *B. bifidum* Z9 or both bacteria in combination. A further analysis of genes within the GO terms resulted in a subdivision of genes encoding for cytokines (interleukins and chemokines) according to the differential stimulation regime, which was not revealed by the gene set enrichment.

Stimulation with *L. acidophilus* NCFM induced a high number of genes involved in pro-inflammatory responses, including activation of the adaptive immune response, e.g. chemokines involved in the attraction of T cells and NK cells [32], whereas other chemokines primarily involved in attraction of cells from the innate immune system, e.g. neutrophilic granulocytes, monocytes and eosinophilic granulocytes, were equally up-regulated after stimulation of DC with *L. acidophilus* NCFM and *B. bifidum* Z9, respectively. Furthermore, the expression of the chemokines strongly up-regulated by *L. acidophilus* NCFM was significantly inhibited when both bacteria were added simultaneously. The same pattern of inhibition was detected for cytokines involved in the stimulation of a Th1 response, e.g. IFN-β, IL-12, IL-18 [13], [33], [34].

Overall, our results show that stimulation of DC with *L. acidophilus* NCFM induces a Th1 polarising response, whereas stimulation of DC with *B. bifidum* Z9 induces a Th2 response. The Th2 polarising profile of DC is not well-described in the literature, but emerging evidence supports that IL-33 and Cxcl1, both up-regulated by *B. bifidum* Z9, play a role in the production of Th2-associated cytokines from *in vitro* polarized Th2 cells and in recruitment of Th2 cells to the target tissue of allergic inflammation [7], [35].

Furthermore, we found that chemokines strongly induced by *L. acidophilus* NCFM were dependent on the up-regulation and subsequent autocrine stimulation of IFN-β. We have demonstrated the same IFN-β dependent mechanism for the gene coding for the Toll-like receptor 3 (TLR-3) (Weiss *et al.*, submitted). Moreover, the expression of a large number of Th1-promoting genes is known to depend critically on stimulation of the cells with IFN-β [13]. Whether the expression of all genes up-regulated only by *L. acidophilus* NCFM and down-regulated by the simultaneous presence of *B. bifidum* Z9 is initiated by IFN-β remains to be elucidated. However, the fact that the most prominent effector mechanisms involved in the antiviral defence are controlled by type I IFNs, in particular IFN-β, indicates that the expression of many genes classified as Th1 inducing may likewise be controlled by IFN-β [14], [36]. IFN-β binds to the type I interferon receptor (IFNAR), an event that initiates the activation of the associated Janus kinases. This in turn activates the IFN-stimulated gene factor 3 (ISG3) via stimulation and heterodimerization of STAT1 and STAT2 [14], [37]. The events leading to the expression of the gene encoding IFN-β upon bacterial stimulation are, at least to some extent, similar to the viral IFN-β-inducing events. The activation of IRF3 seems to be a key point [38]. IRF3 is constitutively expressed in most cells [14], and its phosphorylation is mediated by inhibitors of nuclear factor (NF)-kB kinases. This leads to an association of IRF3 with various transcription factors of the AP-1 family, including ATF-2 and c-Jun, to form an enhanceosome that binds the IFN-β promoter, thus stimulating the expression of the *Ifnβ1* gene [39]. This activation of IRF3 is believed to take place upon activation of TLR4 by Gram negative bacteria through the same mechanisms found for virus induction of IFN-β [40]. Whether a corresponding mechanism for induction of IFN-β upon stimulation with Gram-positive bacteria is existent remains to be established.

Relatively few genes were exclusively up-regulated in DCs stimulated with *B. bifidum* Z9. Notably, the expression of these genes was not affected by addition of *L. acidophilus* NCFM. Thus, whereas *B. bifidum* Z9 has the capability to actively modulate the response in DC induced by *L. acidophilus* NCFM, the opposite does not apply. The expression of the transcription factor *Jdp2* induced in *B. bifidum* Z9 stimulated DC is of particular interest, as JDP2 has been shown to bind to cAMP-response element (CRE) either as a homodimer or a heterodimer with ATF-2 and c-Jun, allowing JDP2 to repress CRE-dependent transcription [41], [42]. ATF-2, and in particular c-Jun, have been shown to stimulate the production of IFN-β. ATF-2 is activated by both JNK and p38, but c-Jun is only activated by JNK [22]. In the present study, we demonstrated that the IFN-β induction is strongly dependent on JNK activation, as inhibition of JNK, but not p38, almost totally abrogated the production of IFN-β and also of the expression of genes and gene products which are dependent on IFN-β, as exemplified in our study with Cxcl10. The up-regulation of *Jdp2* in DC stimulated with *B. bifidum* Z9 indicates that this transcription factor is involved in an active down-regulation of the transcription of the gene coding for IFN-β by changing the composition of the dimer binding to CRE in the enhanceosome, thus inhibiting binding of c-Jun and thereby preventing the transcription of the gene encoding IFN-β. This finding, in combination with the kinetics of expression of the genes encoding IFN-β and IL-12 (Figure 1 and 5), strongly suggests that the up-regulation of *Jdp2* in *B. bifidum* Z9 DC is possibly involved in the inhibition of the stimulatory profile induced by *L. acidophilus* NCFM.

In conclusion, we present data that reveal highly distinct immune stimulating properties of the two probiotic strains *L. acidophilus* NCFM and *B. bifidum* Z9. Our results strongly indicate that *B. bifidum* Z9 employs an active mechanism down-regulating the pro-inflammatory and Th1 skewing profile induced by *L. acidophilus* NCFM in DC.
